# Rare recurrence of a multilocular cystic leiomyoma following myomectomy

**DOI:** 10.20407/fmj.2022-013

**Published:** 2022-10-28

**Authors:** Eiji Nishio, Yoshiko Sakabe, Takuma Fujii

**Affiliations:** Department of Obstetrics and Gynecology, Fujita Health University, School of Medicine, Toyoake, Aichi, Japan

**Keywords:** Pelvic cystic mass, Multilocular cystic leiomyoma, Myomectomy, Infrequent recurrence, Incomplete excision

## Abstract

Multilocular cystic leiomyomas rarely develop following myomectomy. To the best of our knowledge, there are no published reports on recurrent multilocular cystic leiomyoma following myomectomy. We here present such a case. A 45-year-old woman visited our outpatient clinic because of heavy vaginal bleeding. She underwent laparoscopic myomectomy for a solid mass in the uterine cavity. Subsequent pathological examination of the operative specimen revealed a tumour with well-demarcated borders and spindle cells arranged in intersecting fascicles. Seven days postoperatively, ultrasonography revealed a cystic lesion. Magnetic resonance imaging performed 28 months postoperatively revealed a large, well-defined, multilocular cystic mass that was homogeneously hyperintense on T2-weighted images on the exterior of the uterus. Abdominal hysterectomy was performed. On pathological examination of the operative specimen, she was found to have a leiomyoma with marked cystic degeneration. Incomplete excision of a multilocular cystic leiomyoma may result in recurrence in the form of a large cystic mass. Clinical differentiation between a multilocular cystic leiomyoma and an ovarian tumour may be difficult. Complete resection of a uterine multilocular cystic lesion prevents recurrence.

## Introduction

Pelvic cystic masses generally originate in the ovary,^1^ such lesions rarely originating in other pelvic organs. Recurrence of leiomyomas following myomectomy is common.^2^ Herein, we present a rare case of recurrence of a multilocular cystic uterine leiomyoma following myomectomy.

## Case report

**Figure 1:**
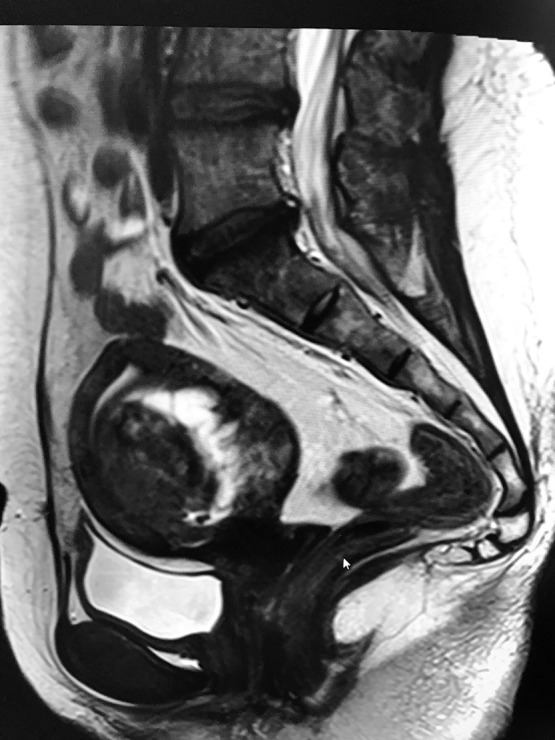
Magnetic resonance imaging (MRI) showing a solid, 60-×50-mm mass with a cystic component within the uterine cavity.

**Figure 2:**
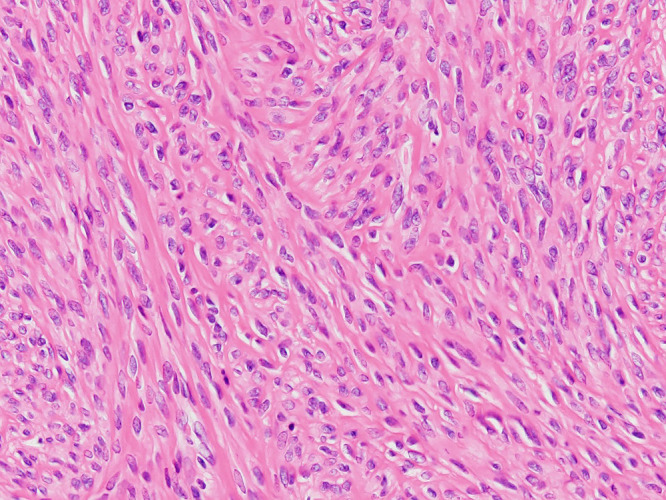
Photomicrograph showing a tumour with well-demarcated borders and spindle cells arranged in intersecting fascicles (200× magnification for each other).

**Figure 3:**
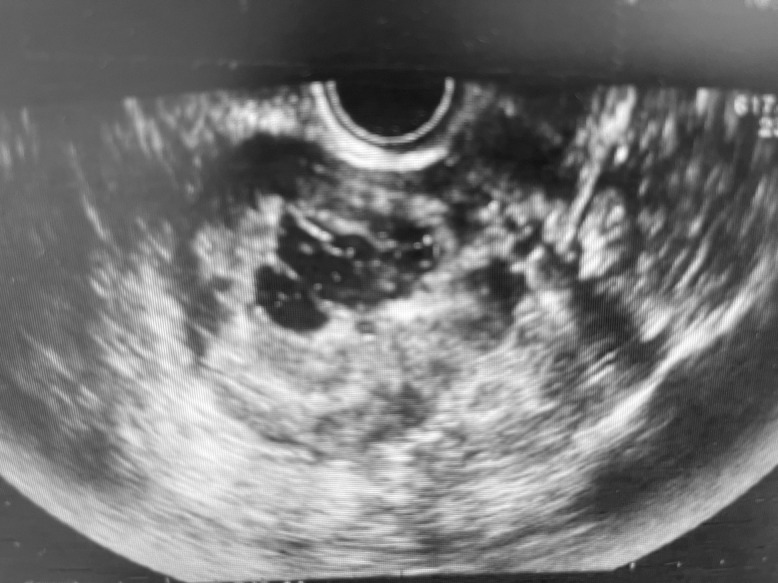
Ultrasound image obtained 7 days postoperatively showing a cystic lesion in the uterine cavity.

A 45-year-old woman (gravida 2, para 2) was referred to our clinic because of heavy vaginal bleeding. Magnetic resonance imaging (MRI) revealed a solid 60-×50-mm mass with a cystic component in the uterine cavity (Figure 1). Laparoscopic myomectomy was therefore performed. During surgery, no intraperitoneal adhesions were observed. The myometrium was incised with an ultrasonic knife and the myomas easily enucleated. The uterine incision was closed in two layers with a needle. Subsequent pathological examination of the operative specimen revealed a tumour with well-demarcated borders and spindle cells arranged in intersecting fascicles (Figure 2). The final histopathological diagnosis was leiomyoma. The patient’s postoperative course was uneventful. Ultrasonography performed 7 days postoperatively revealed a cystic lesion in the uterine cavity (Figure 3). We followed the patient up in the clinic carefully because of the possibility of development of an arteriovenous fistula.

**Figure 4:**
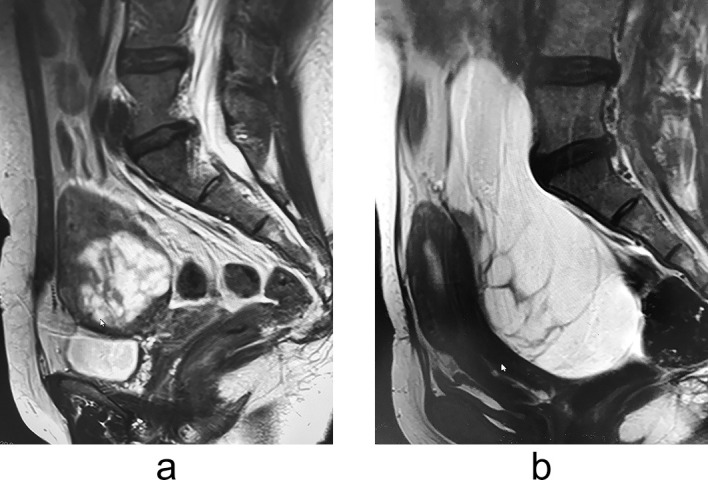
a MRI performed 9 months postoperatively showing a multilocular cystic lesion in the uterine cavity that was homogeneously hyperintense on T2-weighted images. b MRI performed 28 months postoperatively showing a 151-×61-×133-mm, well-defined, multilocular cystic mass on the exterior of the uterus. The lesion was homogeneously hyperintense on T2-weighted images.

**Figure 5:**
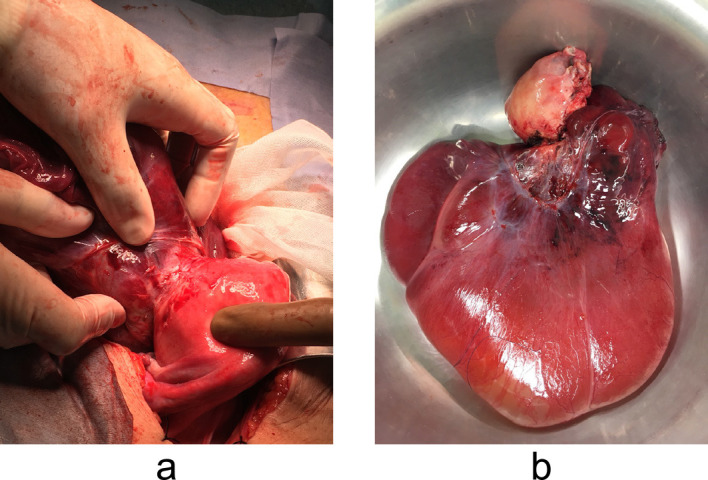
a Intraoperative photograph showing a distended, multilocular, cystic mass protruding from the posterior surface of the uterus. b Photograph showing the cyst had a thick wall.

Nine months postoperatively, the patient visited our hospital because of abdominal distension. MRI revealed a multilocular cystic lesion in the uterine cavity that was homogeneously hyperintense on T2-weighted images (Figure 4a). MRI performed 28 months postoperatively revealed a 151-×61-×133-mm, well-defined, multilocular cystic mass on the exterior of the uterus. The lesion was homogeneously hyperintense on T2-weighted images (Figure 4b). Abdominal hysterectomy revealed a distended multilocular cystic mass protruding from the posterior surface of the uterus (Figure 5a). Part of this mass had been dissected in the previous myomectomy. Both ovaries appeared normal. However, we detected a tumour with a fluid-filled, thick-walled cystic component (Figure 5b). Histological examination revealed a submucosal leiomyoma composed of bland smooth muscle cells growing in broad, sweeping fascicles with no atypical or abnormal cells. This leiomyoma showed marked cystic degeneration.

**Figure 6:**
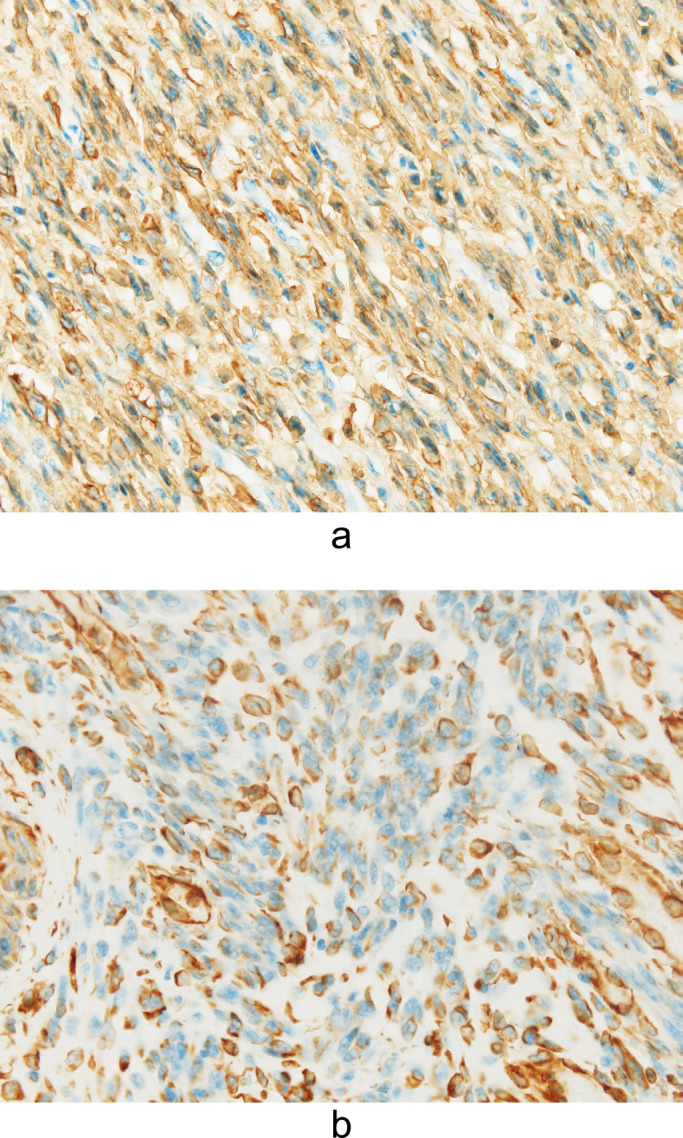
a Photomicrograph showing positive staining for smooth muscle actin (200× magnification for each other). b Photomicrograph showing positive staining for vimentin (200× magnification for each other).

Immunohistochemically, the tissue was positive for smooth muscle actin (Figure 6a) and vimentin (Figure 6b) and negative for cytokeratin AE1/AE3. The final histopathological diagnosis was degenerative leiomyoma.

## Discussion

We here report a rare case of recurrence of a multilocular cystic leiomyoma following myomectomy. Our patient’s clinical course highlights two important points.

First, incomplete excision of a multilocular cystic leiomyoma may result in its recurrence as a large cystic mass. Recurrence of a leiomyoma following myomectomy is common.^2^ However, to the best of our knowledge, there are no previous reports of recurrent multilocular cystic leiomyomas following myomectomy. The differential diagnosis of a multilocular tumour in the uterine cavity includes degenerative leiomyoma, intrauterine endometrial cyst, and arteriovenous fistula. Cyst formation occurs during the late stages of degeneration as hyaline material liquefies.^3^ Endometriosis in the uterine wall has rarely been reported.^4^ The presence of an abnormal uterine vascular network with tortuous and serpiginous vessels is suggestive of an arteriovenous fistula.^5^ In the present case, the absence of blood flow within the cystic lesion excluded this diagnosis. Because the cystic lesion had indistinct borders, we hypothesised that the previous submucosal leiomyoma had been incompletely excised, leading to regrowth of the original lesion.

Second, differentiating between a multilocular cystic leiomyoma and an ovarian tumour may be difficult. The possible differential diagnoses include ovarian tumour, peritoneal inclusion cyst, uterine adenomatoid tumour, and degenerative leiomyoma. One group has reported misdiagnosis of a cystic multilocular leiomyoma as an ovarian tumour.^6^ We did not mistake the lesion for an ovarian tumour in the present case because it was localised in the myometrium adjacent to the site of the previous myomectomy and exhibited exophytic growth. Peritoneal inclusion cysts are complex cystic adnexal masses within which a normal ovary can be enclosed by multiple fluid-filled adhesions. Because peritoneal inclusion cysts develop in the presence of peritoneal adhesions and active ovaries,^7^ this diagnosis should be considered in women of reproductive age who have undergone pelvic surgery. Adenomatoid tumours of the uterus are occasionally misdiagnosed as other benign or malignant neoplasms. Quigley and Hart proposed the following four distinctive histological types: adenoid, angiomatoid, solid, and cystic.^8^

In conclusion, we here present a patient who had a recurrence of a multilocular cystic leiomyoma following myomectomy. It is important to completely resect uterine multilocular cystic lesions to prevent their recurrence.

## References

[1] Moyle PL, Kataoka MY, Nakai A, Takahata A, Reinhold C, Sala E. Nonovarian cystic lesions of the pelvis. Radiographics 2010; 30: 921–938. 20631360 10.1148/rg.304095706

[2] Yoo EH, Lee PI, Huh CY, Kim DH, Lee BS, Lee JK, Kim D. Predictors of leiomyoma recurrence after laparoscopic myomectomy. J Minim Invasive Gynecol 2007; 14: 690–697. 17980328 10.1016/j.jmig.2007.06.003

[3] Kamat NV, Telkar HB, Ramani SK, Thakker AP. Ruptured degenerated uterine fibroid diagnosed by imaging. Obstet Gynecol 2001; 98: 961–963. 11704222 10.1016/s0029-7844(01)01541-1

[4] Yin W, Zhang J, Xu L, Luo L. Intrauterine endometrial cyst after low uterine incision. A case report with literature review. Medicine (Baltimore) 2018; 97: e0376. 29642191 10.1097/MD.0000000000010376 PMC5908554

[5] Barral PA, Saeed-Kilani M, Tradi F. Transcatheter arterial embolization with ethylene vinyl alcohol copolymer (Onyx) for the treatment of hemorrhage due to uterine arteriovenous malformations. Diagn Interv Imaging 2017; 98: 415–421. 27776896 10.1016/j.diii.2016.09.003

[6] Yorita K, Tanaka Y, Hirano K, Kai Y, Arii K, Nakatani K, Ito S, Imai T, Fukunaga M, Kuroda N. A subserosal, pedunculated, multilocular uterine leiomyoma with ovarian tumor-like morphology and histological architecture of adenomatoid tumors: a case report and review of the literature. J Med Case Rep 2016; 10: 352. 27998309 10.1186/s13256-016-1167-1 PMC5175316

[7] Jones SA, Salicco JM, Byers MS. Pelvic pain and history of previous pelvic surgery. Proc (Bayl Univ Med Cent) 2003; 16: 121–122. 16278726 10.1080/08998280.2003.11927892 PMC1200814

[8] Quigley JC, Hart WR. Adenomatoid tumors of the uterus. Am J Clin Pathol 1981; 76: 627–635. 7293978 10.1093/ajcp/76.5.627

